# Ultrafast Coherent Delocalization Revealed in Multilayer QDs under a Chiral Potential

**DOI:** 10.1021/acs.jpclett.2c03743

**Published:** 2023-02-23

**Authors:** Hanna T. Fridman, Hadar Manis Levy, Amitai Meir, Andrea Casotto, Rotem Malkinson, Joanna Dehnel, Shira Yochelis, Efrat Lifshitz, Nir Bar-Gill, Elisabetta Collini, Yossi Paltiel

**Affiliations:** † Applied Physics Department, Jerusalem, The Hebrew University of Jerusalem, Jerusalem 91904, Israel; ‡ Department of Chemical Sciences, University of Padova, Via Marzolo 1, I-35131 Padova, Italy; § Nancy and Stephen Grand Technion Energy Program, Russell Berrie Nanotechnology Institute, Quantum Information Center, Schulich Faculty of Chemistry, Solid State Institute, Technion Israel Institute of Technology, Solid Stat, IL-3200003 Haifa, Israel; # The Racah Institute of Physics, The Hebrew University of Jerusalem, Jerusalem 91904, Israel; @ The Center for Nanoscience and Nanotechnology, The Hebrew University of Jerusalem, Jerusalem 91904, Israel

## Abstract

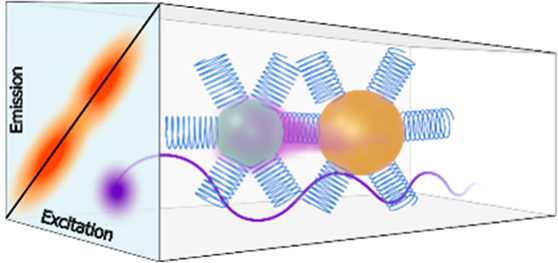

In recent years, it was found that current passing through chiral molecules exhibits spin preference, an effect known as Chiral Induced Spin Selectivity (CISS). The effect also enables the reduction of scattering and therefore enhances delocalization. As a result, the delocalization of an exciton generated in the dots is not symmetric and relates to the electronic and hole excited spins. In this work utilizing fast spectroscopy on hybrid multilayered QDs with a chiral polypeptide linker system, we probed the interdot chiral coupling on a short time scale. Surprisingly, we found strong coherent coupling and delocalization despite having long 4-nm chiral linkers. We ascribe the results to asymmetric delocalization that is controlled by the electron spin. The effect is not measured when using shorter nonchiral linkers. As the system mimics light-harvesting antennas, the results may shed light on a mechanism of fast and efficient energy transfer in these systems.

The coupling between sites in different light harvesting biological systems has been studied intensively over the past few years. In these studies, the harvesting efficiency was probed for different coupling conditions.^1−4^ These include temperature, pressure, the distance between sites, and excitation energy.^5−7^ Since many biological molecules and systems are chiral,^8−10^ and chiral coupling between two excited states differs,^11^ it would be interesting to probe the coupling in the short time scale where coherent effects exist in the chiral system. This is the core of the study in the presented work.

In recent years it was found that current passing through chiral molecules exhibits spin preference, an effect known as the Chiral Induced Spin Selectivity (CISS) effect.^12−14^ It is known that this spin-filtering effect may affect the coupling between semiconductor nanocrystals (Quantum Dots, QDs). Indeed, it was shown that for samples of CdSe heterosized QDs, coupled via chiral linkers, an enhancement in the electron wave function delocalization as a function of the induced excited spin direction was observed.^11^ In other words, when the excited spin direction agrees well with the organic linker chirality, the coupling strength of the neighboring QDs increases, facilitating energy distribution between the dots even through a long chiral molecule (2.2 nm). Moreover, it was shown in a layered device that when chiral-bound QDs are illuminated with circularly polarized light (CPL), charge separation can occur, depending on the excited spin chirality.^15,16^ We study here the coupling in similar artificial chiral systems in ultrafast time regimes, where coherent and quantum effects might appear and be affected by the chiral nature of the samples.

In order to mimic the light-harvesting systems, we followed the design already proposed in previous works.^5,6^ In these works, QDs having discrete energy levels due to quantum confinement are mimicking the chromophores, while the environment is generated using organic linkers. In the current work, about 28 layers of QDs are self-assembled on a quartz substrate to achieve a semiordered structure with an optical density of 0.1 that can be probed by ultrafast spectroscopic techniques. The samples were assembled on the substrate using wet chemistry, where L α-helix polyalanine chiral helical molecules ([H]-C(AAAAK)_7_-[OH]) covalently linked the QDs monolayers/multilayers. To show that chiral linkers and excited spin play a role on the fast time scale, an additional fluorescence quenching experiment was done using a monolayer of QDs deposited on a gold substrate. Building upon previous results^17−20^ demonstrating the presence of coherent effects with very short organic linkers, we wanted to probe the possible effects of the coupling promoted by longer chiral linkers at similar short time scales. This was achieved using two-dimensional electronic spectroscopy (2DES),^21−23^ a technique already proven to be particularly useful in the investigation of coherent dynamics in strongly coupled systems.^17,24,25^ The investigation was done on semiordered multilayers prepared by deposition of one- or two-sized donor–acceptor CdSe QDs (see Methods section in the Supporting Information).

Figure 1 presents the two types of samples explored in this study. The 2DES samples (Figure 1a) are constructed using multilayers of QDs/chiral linkers that are achieved via layer-by-layer self-assembled adsorption, creating an aggregated structure of CdSe QDs with chiral linkers in between. This structure ensures the high optical density needed for the 2DES measurements. As a chiral linker, we used the helical molecule L α-helix polyalanine, 36-amino-acids, ∼5.4 nm in length,^26^ which exhibits a monolayer height of 4.3 nm.^15^ Two different sizes of the QDs were chosen to achieve a good coupling between the 1S level of the donor and the 2S level of the acceptor.^6^ The diameter of the two different QDs was ∼2.8 nm (small, S) and ∼3.5 nm (big, B). Figure 1b presents instead the QDs that are self-assembled to a monolayer on a gold-evaporated substrate. In this experiment, the fluorescence quenching efficiency for different linkers was tested for two circular polarization excitations. Also in this case, the L α-helix polyalanine was used as a linker. For both experiments, QD samples assembled with achiral organic linkers were also prepared in analogous ways and studied as a reference. More details on the samples’ preparation are provided in the Supporting Information (SI), in section S1 and Figures S1 and S2.

**Figure 1:**
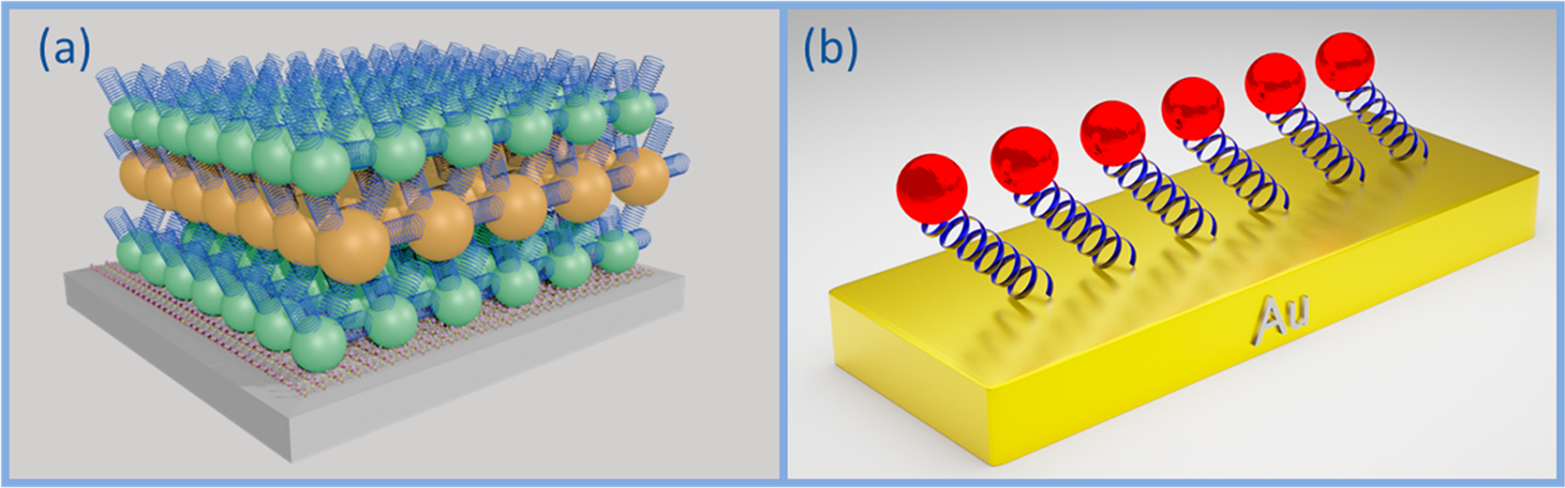
(a) A schematic illustration of the multilayers QD sample constructed via layer-by-layer self-assembled adsorption of QDs and chiral linkers (L α-helix polyalanine, 36 amino acids) studied in 2DES measurements; the final result is an aggregated structure of CdSe QDs with chiral linkers in between. (b) A schematic illustration of the QDs self-assembled monolayer on a gold evaporated substrate studied in fluorescence quenching experiments. In both cases, control samples with the same design but using achiral linkers have also been prepared.

In the 2DES experiments, the response of the chiral multilayer sample illustrated in Figure 1a (labeled from now on as MixC) was compared with the response of a series of achiral samples. In order to explore a set of reference samples as complete as possible, we considered achiral multilayers in different coupling conditions, acting on the length of the linkers and on the size of the dots. First, we considered achiral “mix” samples prepared by alternate deposition of layers of big and small dots, like for the chiral sample, but using two organic achiral linkers with shorter lengths: 1,3-propandithiol (O3), ∼0.57 nm in length,^27^ and 1,9-nonanedithiol (O9), ∼1.3 nm in length.^28^ We named these two samples MixO3 and MixO9, respectively. In addition, we also analyzed “homo” samples, characterized by coupling between dots of similar size, either small or big dots, prepared using the shortest O3 linker. These two samples are denoted as HomoS and HomoB, respectively.

Figure 2 presents the extinction spectra of the measured samples. Schematic representations of the samples are shown in the insets, and an illustration of the linkers is shown next to the sample name. The gray area shown in Figure 2a depicts the laser spectral profile used for the excitation in the 2DES experiments (more details about the 2DES experiment in SI, section S2 and Figure S3). Figure 2a and b emphasize the matching between the 1S and 2S levels of the small and the big QDs, respectively. The extinction spectra of the Mix samples (see Figure 2c and d) indicate a more efficient mixing of the two kinds of dots in the MixC sample than in the other two samples.

**Figure 2:**
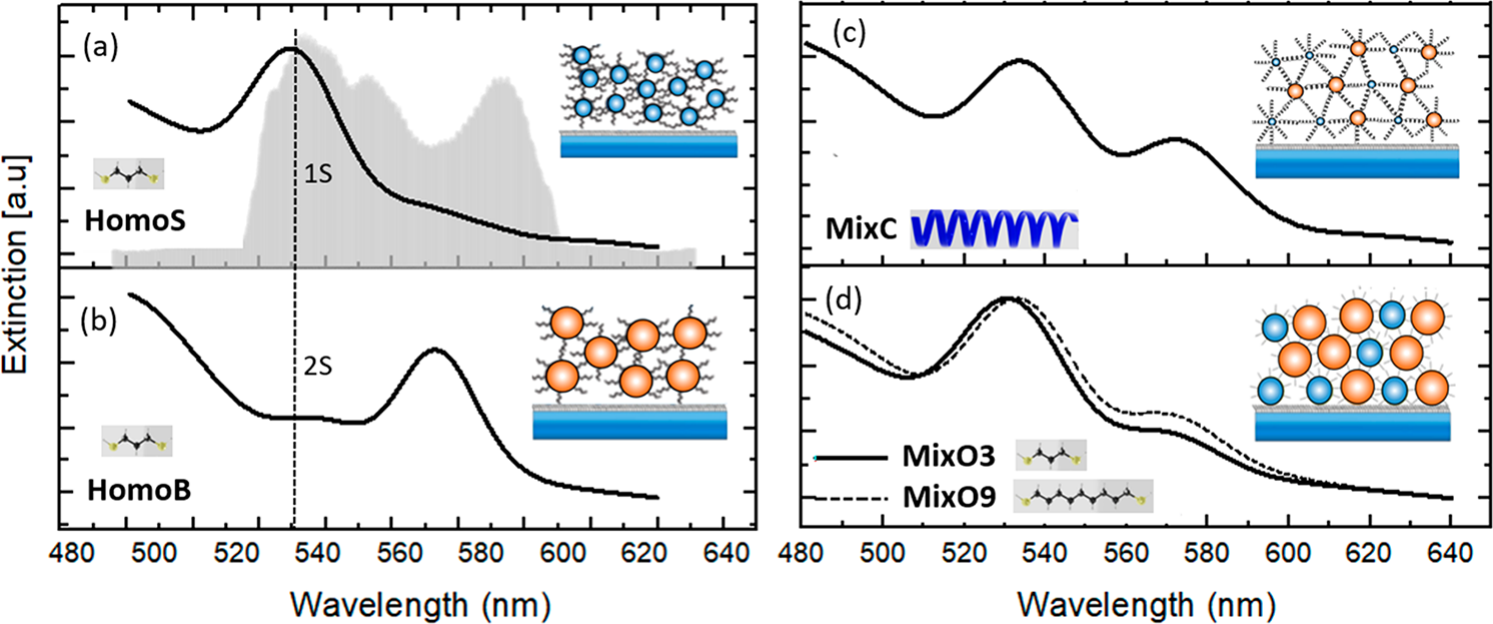
Extinction spectra of the five QD multilayer samples studied by 2DES: (a) HomoS; (b) HomoB; (c) MixC; (d) MixO3 and MixO9. In each panel, a schematic of the sample and an illustration of the linker molecules are shown. The gray area in a depicts the laser spectral profile used for the excitation in the 2DES experiments. The matching between the 1S energy level and the 2S energy level of the small and big QDs is emphasized by the dashed line shown in a and b. A comparison between c and d spectra reveals a more efficient mixing process for the MixC sample than for the two MixO samples, indicating a greater amount of adsorbed big QDs in the case of the MixC sample.

Examples of the rephasing, non-rephasing, and purely absorptive 2DES maps at selected values of population time *t*_2_ for the five samples are presented in the SI (Figures S4–S8). The 2DES are analyzed with a global complex multiexponential fit method as proposed in ref (29). The 2DES signal decay can be well fitted with three time constants: (i) an ultrafast time constant on the order of tens of femtoseconds that can be attributed to ultrafast processes such as hot carrier relaxation, spectral diffusion, and scattering phenomena; (ii) a second component associated with intra/interdot relaxation and/or a surface related relaxation channel due to the presence of thiol ligands; and (iii) a long time component (≫1 ps) describing the overall decay of the maps following relaxation processes happening on time scales well beyond the investigated experimental time window. All of these dynamic processes have already been documented in the literature.^30−36^ While the first and the third kinetic components have the same dynamics and amplitude distribution among the five samples, the second time component presents significant differences, as summarized in Figure 3, where the decay-associated spectra (DAS) of the second time constant for the samples considered in this work are reported.

**Figure 3:**
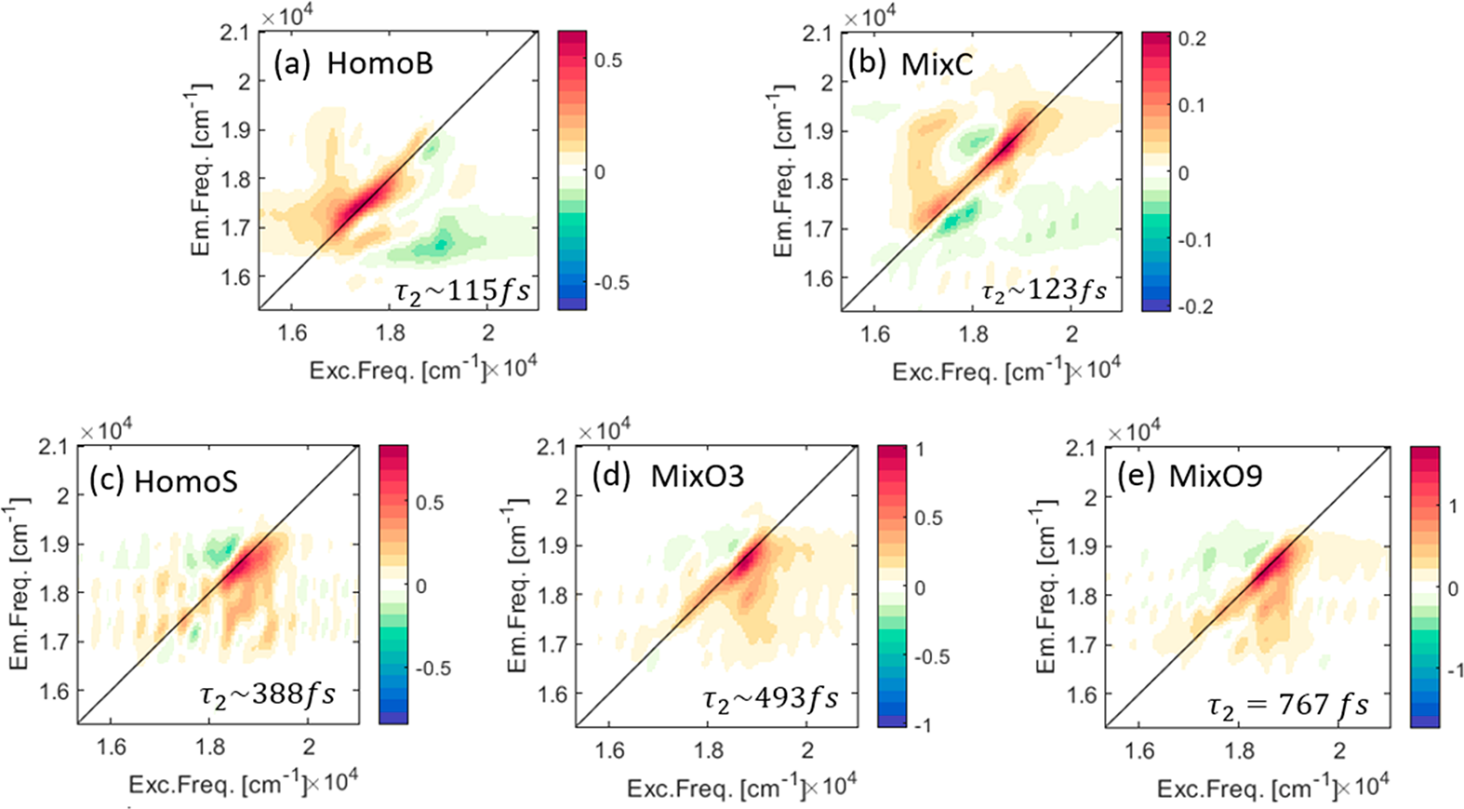
DAS maps of the five QD multilayer samples: (a) HomoB, (b) MixC, (c) HomoS, (d) MixO3, and (e) MixO9. On the bottom of each map, the second time constant is written. Samples HomoB and MixC show an extended negative signal (green) below the diagonal and shorter relaxation times in comparison to the other samples, interpreted in terms of an interdot energy transfer from high to low frequency states.

A DAS map shows the amplitude distribution associated with a particular time constant in a 2D map, as emerging from the global fitting. The color spectrum of the peaks in a DAS depends on whether the signal is defined by exponentials with a positive or negative amplitude. In cases where a signal in the original 2D map has a positive amplitude, a corresponding positive (negative) peak in a DAS at the same coordinates implies that, overall, the signal at those coordinates is decaying (rising). All of the DAS in Figure 3 present a red positive signal in correspondence with the main diagonal peaks, suggesting a decay of the population of the excited states. In addition, the DAS of both the HomoB and the MixC samples show an extended negative signal (green) below the diagonal, not shown in the other samples, suggesting that at these coordinates the signal is rising. Such a peculiar signal distribution is typically attributed to relaxation from higher to lower energy states, as already documented for other systems.^37−40^ This peculiar signal distribution in the DAS supports the attribution of the second time constant to intra/interdot relaxation channels, as suggested before. There are two possible dynamic processes that can justify a similar behavior: (i) interdot energy transfer from small to big QDs^6^ or among delocalized states of interacting big QDs^17^ and (ii) intradot relaxation from the 2S to the 1S energy level of the big dots.^20^ It is reasonable to assume that these processes would give rise to similar rising (green negative) signals at the same below diagonal coordinates because of the resonance between the 2S of the big dots and the 1S of the small dots. It is important to note that the excitation wavelength range of our measurement system allows only the 1S level of the small quantum dots (QDs) to be excited.

The presence of a possible contribution of the intradot process (ii) seems to be fostered by previous measurements on samples of monomeric QDs in a solution having a similar size to the HomoB sample and showing a DAS with an analogous signal distribution.^20^ However, in the previous case, a longer relaxation time was associated with this DAS (197 and 305 fs, see ref (20)’s SI). This suggests that, while a contribution of intradot phenomena cannot be completely ruled out, a strong interdot transfer component (i) should be taken into account to justify our findings for HomoB and MixC samples. This hypothesis is also supported by the beating analysis, as will be discussed later. A similar trend, although expected also for the HomoS, MixO3, and MixO9, was not observed for these samples. According to the HomoS sample absorption spectrum presented in Figure 2a, only the 1S state is excited by the system laser’s wavelength range; thus, only interdot interactions are expected for the HomoS. Also, although expected, such a trend was not captured in MixO3 because the mixing process of the two size QDs was not efficient as in the MixC sample (as observed in the extinction spectra in Figure 2c and d). Concerning MixO9, even though the mixing process was achieved more efficiently than in the MixO3 case (see Figure 2d), it is likely that the long distance between the dots due to the longer linker length prevented the efficient delocalization of the excitation among dots.

In addition, the relaxation time constants of the HomoB and the MixC samples are relatively shorter than in the other samples. MixO3 and MixO9 are characterized by longer relaxation times than in the case of the HomoB and MixC samples and might imply a dominance of intradot transitions. Samples HomoS and MixO3 share more close relaxation time values due to the similarity of their transitions characterized by inter-small-dots transitions, as noted before that the MixO3 sample has less efficient mixing. Note that the MixO3 sample has a longer relaxation time as it contains also a strong contribution of intradot transitions. Sample MixO9 shows the longest relaxation time of ∼767 fs. It is reasonable to assume that in this sample, due to the larger distance between the dots, interdot phenomena are negligible, and the typical intradot behavior found for monomeric samples in solution is predominant. Faster relaxation times for interdot than for intradot transitions stand in analogy to faster relaxation times for intermolecular transitions in interacting multichromophoric samples characterized by delocalized excitations.^41,42^ This can also explain the decreased relaxation time when moving from monomeric samples to interacting samples discussed in the previous paragraph.

In order to support our interpretation, we performed an analysis of the coherent beating behavior appearing in the 2DES signal at early times. In previous work, this analysis, supported by theoretical modeling, already revealed significant insight into the intra- or interdot nature of the captured phenomena.^17^ Building on these previous results, time–frequency transform (TFT) analysis was applied,^43,44^ which allows a more direct way to visualize the frequency and dephasing times of beating modes. This approach overcomes the limitations of conventional methods based on Fourier transforms, maintaining both frequency and time resolution. A TFT spectrum, indeed, shows, on the ordinate, the frequencies of the components contributing to the beating pattern at a specific coordinate of the 2D map, like a conventional Fourier spectrum, and on the abscissa, their time evolution.^43^

Figure 4 presents the results of the TFT applied for the HomoB and MixC samples to the signal extracted at off-diagonal coordinates as shown in panel a. TFT plots for the other samples are reported in the Supporting Information (Figure S9). Below-diagonal off-diagonal coordinates are particularly relevant in the quest for interdot phenomena, as recently demonstrated by theoretical predictions.^45^ TFT maps at relevant coordinates for the homointeracting QD samples were already discussed in ref (17) and were supported by theoretical simulations. In agreement with these previous results, the beatings between 500 and 1500 cm^–1^, shown for the HomoB sample, can be associated mainly with interdot coherences. Beatings below 500 cm^–1^ are instead associated mainly with a longitudinal optical (LO) phonon and intraband transition that have already been characterized in several CdSe QD samples in the literature.^17,20,34,46,47^ The TFT map of the MixC sample shows exceptional similarity to the map of HomoB, with several high intensity beating modes with a frequency above 500 cm^–1^. The beating behavior is instead completely different for the other samples (see Figure S9 in the Supporting Information). The beating pattern in the TFT plots can be used to gain insights into the energy band structure, since the beating frequencies (on the *y*-axis) reflect energy gaps among states, as well as on the dephasing times of coherent superpositions.^45^ On the one hand, the similarity of the beating pattern of MixC with HomoB suggests an analogy between the electronic structure of the two samples. This evidence can be justified considering that the 1S energy level of the small QDs matches the 2S energy level of the big QDs, which can promote a possible interdot delocalization of the excitation among different QDs. On the other hand, the TFT plots show that the coherent beatings last for a time significantly longer than the pulse duration, supporting the hypothesis that interdot coherent phenomena develop in our systems in a time range of hundreds of fs. Overall, the TFT analysis supports the presence of interdot dynamic phenomena and confirms the interpretation emerging already from the DAS analysis, as reported above.

**Figure 4:**
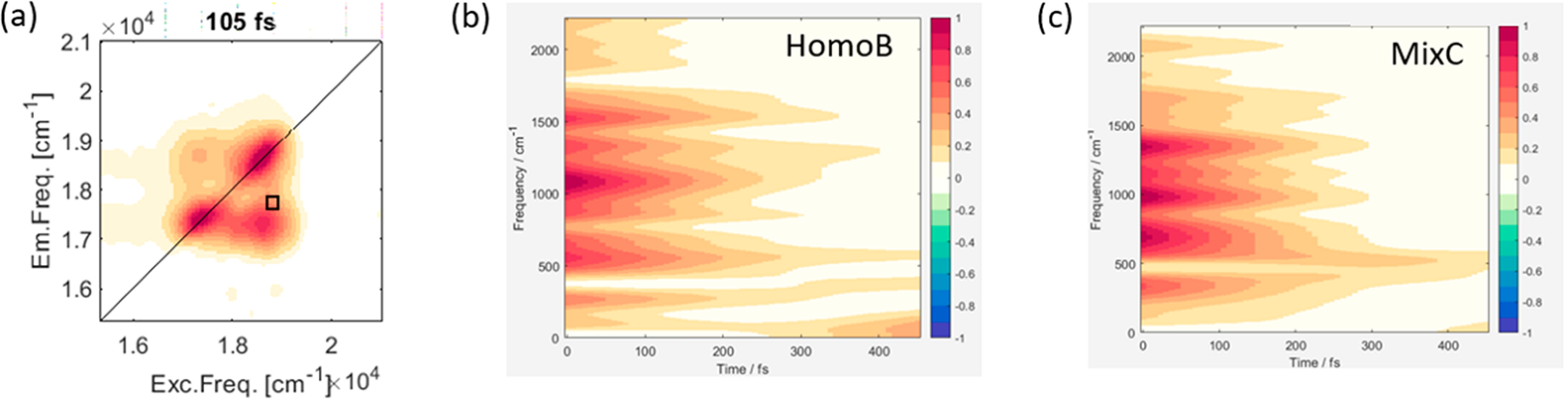
TFT plots of the signal extracted at the off-diagonal coordinates as shown in panel (a) for the HomoB (b) and MixC (c) samples. Sample MixC shows beatings with a pattern similar to the HomoB sample in the frequency range between 500 and 1500 cm^–1^, which indicates interdot transitions.

The most important finding emerging from the analysis of the dynamics of the MixC sample, also in comparison with the other samples containing interacting QDs, is that both population and coherent dynamics are compatible with the presence of strong coupling between the dots, which promotes interdot delocalization. While the presence of interdot coupling has already been experimentally and theoretically demonstrated in samples where QDs are separated by short linkers (like in HomoS and HomoB samples), an indication of interdot transitions in the MixC sample is surprising because of the significant long distance between QDs (∼4.3nm). We associate the strong coupling in this sample to an effective mixing process, energy level matching, and a possible large delocalization of the excited electron through two QDs or more, which might originate thanks to the CISS effect.

In order to relate the coupling between dots with long chiral linkers to a spin effect, a fluorescence quenching experiment was done where a gold substrate serves as a quencher (see Figure 1b). In this experiment, the dynamics in the 300 ps to 10 ns time window were probed (see SI, section S3 for more information). The spin-dependent coupling under chiral potential was studied by fluorescence lifetime measurements using circularly polarized light (CPL). The samples are excited with right/left CPL (R-CPL and L-CPL, respectively) to produce initial different spin states within the dots. The decay time depends on the quenching probability, thus providing spin-dependent coupling information. Figure 5 displays the lifetime values that were extracted from the decay data (shown in the inset) analysis. For chiral linkers, a shortening of the lifetime is revealed when excited by L-CPL compared to R-CPL. These results demonstrate that when exciting with L-CPL, the coupling to the substrate is stronger. Note that there is a negligible difference between L-CPL and R-CPL illuminations using nonchiral molecules. With nonchiral linkers, the measured lifetime value is between the two-lifetime values measured for chiral linkers under different circular polarizations. These differences indicate a dissimilarity in the coupling that is controlled by the excited electron spin under a chiral potential.^11,15,16,48,49^

**Figure 5:**
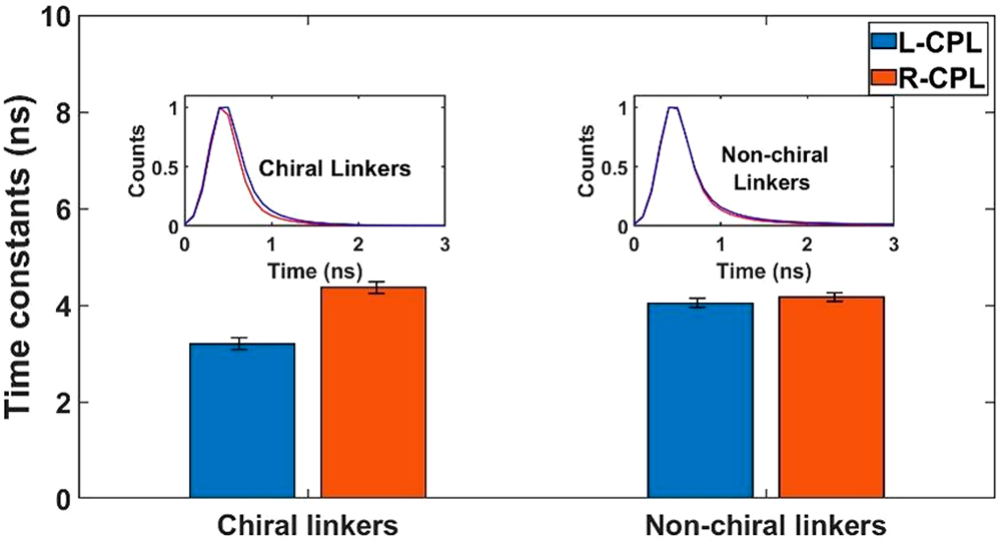
Lifetime values extracted from the decay fitting of the quenching experiment for the excitations with L-CPL (in blue) and R-CPL (in red). Inset: The detected L-CPL (red) and R-CPL (blue) photoluminescence decays.

Summarizing all of the above results, a model (Figure 6) is suggested that accounts for the observations utilizing previous results^11^ and the CISS effect.^13−16,50^ Two major key points are addressed, starting with the population transfer observed in the corresponding 2D DAS maps and carrying on with the short time scale of the second DAS component of the MixC sample. The fact that the population transfer was observed for the MixC sample with a large distance between dots seems to imply that the chiral structure enhances coupling and energy transfer. As mentioned earlier, coupling by chiral linkers depicts a combination of CISS-mediated processes: under a given circular polarization, the chiral potential spreads the wave function of the electron–hole couple in opposite directions toward the adjacent dots, creating a bidirectional unsymmetrical delocalization. This antisymmetric delocalization is large and depends on the direction of the excitation as well as the combination of left and right circular polarizations, generating a spread function that can be measured by symmetry breaking of the sample. Considering that the sample was assembled layer by layer on the surface, generating a semiordered “crystalline” configuration with linking between big–small QDs, we expect that any direction of delocalization will couple big and small dots. As a result, overall enhancement of coupling occurs for both spin directions. In other words, the charge separation causes the center of the wave function of each of the charge carriers to further tend toward the corresponding wave function’s spreading direction,^15,16^ consequently increasing the overlap of the energy states between the adjacent dots on both sides in a rachet kind of mechanism at the same time.

**Figure 6:**
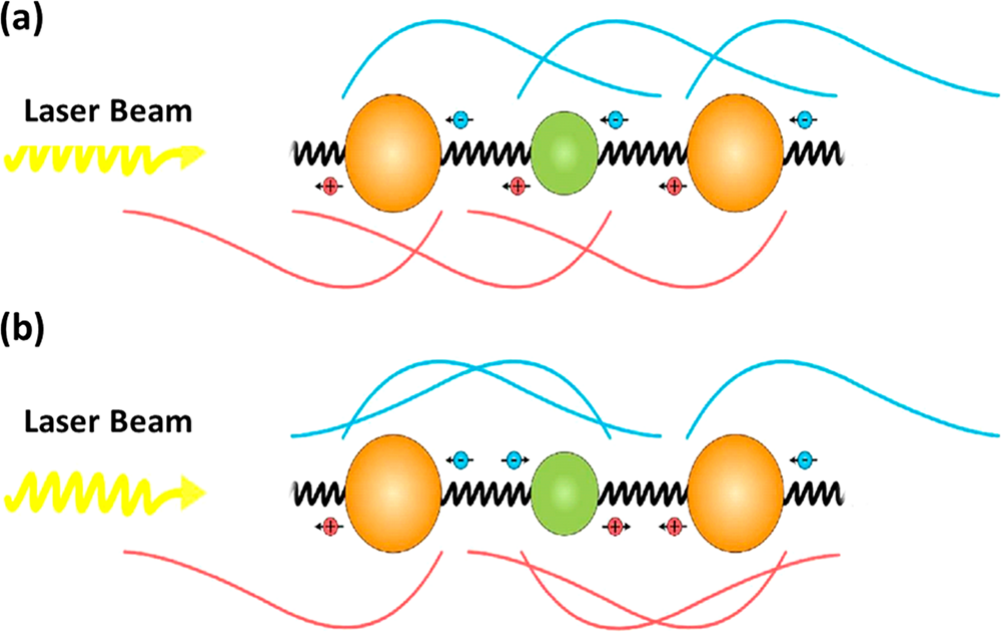
Schematic illustration of the delocalization and coupling mechanism in two evenly possible cases. (a) Charge carriers of neighboring QDs sharing the same spin. The wave functions of the electrons and the holes are delocalized unsymmetrically to the same side, strengthening the coupling with the adjacent QDs. (b) Charge carriers of neighboring QDs with opposite spins. Strong coupling is achieved via high overlapping of the wave functions of the charge carriers of the QD with the ones of the two adjacent QDs.

In the 2DES measurements, the three pulses interacting with the sample have linear polarization, containing a superposition of right- and left-handed circular polarizations, thus exciting even amounts of both spins. Either way, the linear polarization of the pulses increases the overlap of neighboring QDs in both ways, subsequently increasing the coupling strength of the dots in the sample. The proposed model is schematically illustrated in Figure 6, depicting the unsymmetrical delocalization of the charge carriers upon the QD film in two cases. Figure 6a presents the case where the adjacent dot shares the same spin, and Figure 6b presents the case where adjacent dots have a different spin.

This alone cannot explain the MixC small, second time constant. For a proper understanding of the connection between the above model and this short time scale, the subject should be taken into a larger context. The appearance of a novel dynamic on such a short time scale (123 fs) is outstanding, especially when compared to the MixO9, for which the second component time constant is over 6 times larger (767 fs). This difference is even more exceptional when considering that the coupling between dots was found to decay with the linker’s length^6,17^ and that the chiral linkers are 4 times longer than the nonchiral ones (4.3 nm vs 1.3 nm).

The CISS mediated delocalization described earlier could stand at the origin of this phenomenon. Delocalization within ∼200 fs, and even down to ∼60 fs, has been found to happen in biological systems of self-assembled dimers in the strong coupling regime.^51−53^ There is also the possibility of fast energy transfer that is enhanced by coherent chiral phonons.^54^

Indeed, the common denominator of these results and ours is the presence of chiral molecules: the vast majority of biological molecules share chirality,^8−10^ and the molecules that were in use in those experiments are no exception. It is reasonable to assume that these fast timescales are a direct outcome of the CISS effect, where the chiral potential enables a strong coupling mechanism between two chromophores under random polarized or nonpolarized light. The mentioned observations consolidate a notion that quantum effects or, more specifically, spin-related effects play a major role in a vast number of biological phenomena.

The system represented in MixC sample aspires to mimic a quantum-biological system by combining two sizes of QDs resonant in transition energies via chiral molecules as linkers. We demonstrate in our work an ultrafast energy transfer analogous to the one found in biological systems.

A hybrid multilayered QD with a chiral polypeptide linker system was studied here using fast spectroscopy. The delocalization of the exciton generated in the dots is not symmetric and relates to the electronic and hole excited spins. This asymmetric delocalization enables very fast ratchet-like energy transfer between dots of different sizes. The effect is not measured when using shorter nonchiral linkers. As the system mimics light-harvesting systems, the results may shed light on a mechanism of fast and efficient energy transfer in these systems.

## Supporting Information Available

Additional details about the samples’ preparation, additional details about 2DES measurements, additional details about fluorescence quenching experiments (PDF)
