# Supplementary material for: Ultrafast Coherent Delocalization Revealed in Multilayer QDs under a Chiral Potential
Source: J Phys Chem Lett. 2023 Feb 23;14(8):2234–40. doi: 10.1021/acs.jpclett.2c03743 (PMC11139383; doi:10.1021/acs.jpclett.2c03743)
Supplement: Supplementary file 1 — jz2c03743_si_001.pdf [file jz2c03743_si_001.pdf]

Ultrafast Coherent Delocalization Revealed in Multilayer QDs Under a Chiral Potential - Supporting Information

Hanna T. Fridman[1+], Hadar Manis Levy [2+], Amitai Meir [1], Andrea Casotto [2], Rotem Malkinson [3], Johanna Dehnel [4], Shira Yochelis [1], Efrat Lifshitz [4*], Nir Bar-Gill [3*], Elisabetta Collini [2*], and Yossi Paltiel [1*]

[1, 3] Applied Physics Department, Jerusalem, The Hebrew University of Jerusalem, Jerusalem 91904, Israel

[2] Department of Chemical Sciences, University of Padova, Via Marzolo 1, I-35131 Padova, Italy

[3] Technion Israel Inst Technol, Nancy & Stephen Grand Technion Energy Program, Quantum Informat Ctr, Schulich Fac Chem, Solid ,Russell Berrie Nanotechnol Inst Stat, IL-3200003 Haifa, Israel

List of Content:

S1. Additional details about the samples' preparation:

S1.1 Quantum Dots Synthesis.
S1.2 Quantum Dot Extinction Spectra.
S1.3 Multilayers' preparation.

S2. Additional details about 2DES measurements

S2.1 Experimental Setup

S2.2 Additional 2DES maps

S2.3. Additional TFT analysis.

S3. Additional details about fluorescence quenching experiments

S1. Additional details about the samples' preparation

S1.1 Quantum Dots Synthesis

The CdSe QDs with different sizes were prepared according to the procedure given below. In general, a mixture of cadmium oxide (CdO) and oleic acid (OA) in a molar ratio of 1:4 and 7.5 mL of 1-octadecene (ODE) was put in a 25 mL three-neck flask. The reaction mixture was degassed for 1 h at 100 °C under vacuum. Under nitrogen, the temperature was then raised to 300 °C until the solution turned clear, indicating the formation of cadmium oleate. Then the solution was cooled, and amine was added in a molar ratio of 1:8 (Cd/amine). Depending on the reaction, either hexadecylamine (HDA) or octadecylamine (ODA) was used. Afterward, the solution was heated to the desired temperature and trioctylphosphine selenide (TOPSe) solution was injected under vigorous stirring. The growth was terminated after given reaction time by rapid injection of 10 ml of ODE and the reaction mixture was further cooled down by water bath. As-prepared core CdSe QDs were precipitated twice with a 2-propanol/ethanol mixture (1:1−1:2), separated by centrifugation, and dissolved in hexane.

S1.2 Quantum Dots Extinction Spectra

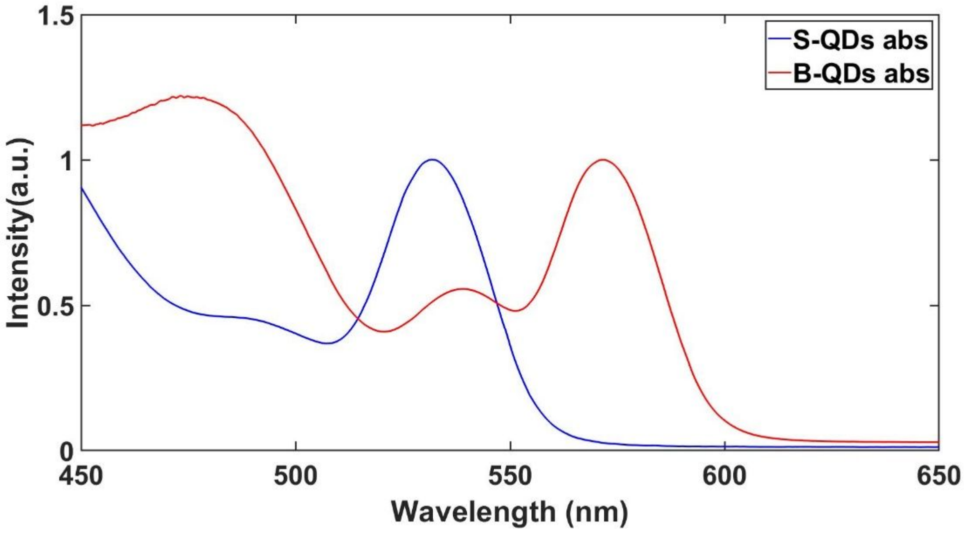

Figure S1. The extinction spectrum of the two QDs used to prepare the multilayer samples in a hexane solution. The S-QDs have a diameter of 2.8 nm and a 535 nm max absorption peak. The B-QDs have a diameter of 3.5 nm and a 570 nm absorption peak

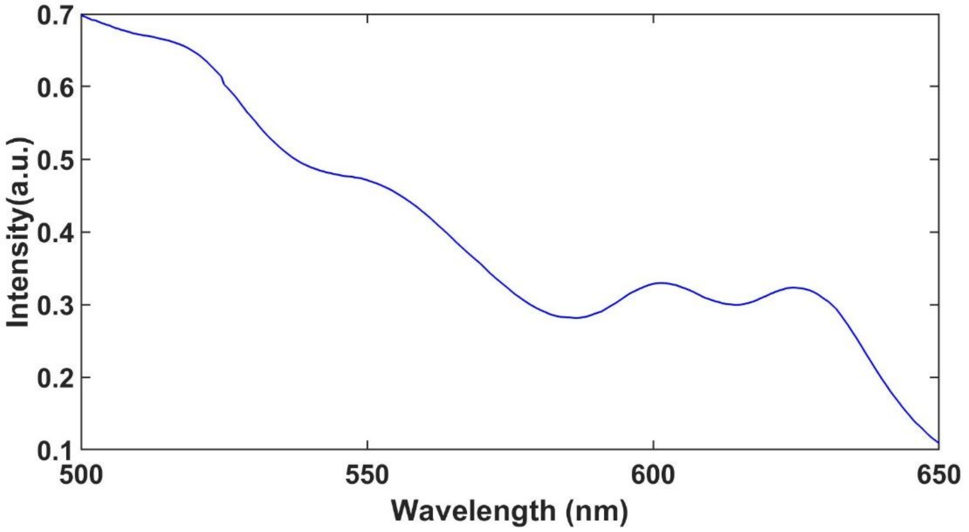

Figure S2. The absorption spectrum of the QDs used to prepare the monolayer samples in a hexane solution. The QDs have a diameter of ~4.5 nm and a 625 nm max absorption peak.

S1.3 Multilayer Preparation

For the multilayer structure, layer-by-layer adsorption by dipping fused silica substrates alternatively between QDs and molecules solutions, as described in more detail elsewhere[1,2] was utilized to achieve an aggregated structure of CdSe QDs (synthesized at Efrat Lifshitz's lab) . The smaller QDs were chosen with a mean diameter of about 2.8 nm, 535 nm absorption peak (figure S1), and the larger ones with a mean diameter of 3.5 nm, 570 nm absorption peak (figure S1). The multilayers were covalently bound by the L-α-helix polyalanine, 36-amino-acids chiral linker (see main paper, Figure 2c), which is 5.4 nm in nominal length (purchased from Merck Israel Inc.), and by non-chiral linker, 1,3-nonanedithiol, 1,9-nonanedithiol (see main paper ,Figure 2d) which is 0.57 and 1.3 nm in nominal length, respectively, (purchased from Merck Israel Inc). For the homostructures; single-sized QDs were used and linked by the 1,3-nonanedithiol (see main paper, Figure 2a,2b). Disordered structures of covalently bonded QDs were realized by dipping the fused silica substrates alternatively between the different QDs. The process was repeated ~28 times, until an optical density of about 0.1, which is necessary for the nonlinear measurements, was obtained. To prevent oxidation, the adsorption process was carried out under a nitrogen environment, and the samples were immediately sealed by evaporated 20 nm aluminum oxide layer, followed

by encapsulation between two fused silica substrates using Norland Optical Adhesive 61 (NOA61).

For the monolayer structure, CdSe QDs having main absorption peak at 625 nm (figure S2), were covalently linked by chiral\non chiral molecules to evaporated Au substrates. L-α-helix polyalanine ([H]-C(AAAAK)$_7$-[OH]),36-amino-acids (purchased from Merck Israel Inc.) was used as the chiral linkers (see main paper, Figure 1b) and 1,11-undecandithiol was used for the non-chiral linkers (1.5 nm in length).

S2. Additional details on 2DES measurements

S2.1 Experimental setup

2DES measurements were performed in the fully non-collinear BOXCARS geometry using the setup described in Ref[3]. Briefly, the output of an 800 nm, 3kHz Ti:Sapphire laser system (Coherent Libra) is converted into a broad visible pulse in a non-collinear optical amplifier (Light Conversion TOPAS White). The transform-limited condition for the pulses at the sample position is achieved through a prism compressor coupled with a Fastlite Dazzler pulse shaper for the fine adjustment. The 2DES experiment relies on the passively phase stabilized setup, where the laser output is split into four identical phase-stable beams (three exciting beams and a fourth beam further attenuated of 3 orders of magnitude and used as Local Oscillator, LO) in a BOXCARS geometry using a suitably designed 2D grating. Pairs of 4° CaF_2 wedges modulate time delays between pulses. One wedge of each pair is mounted onto a translation stage that regulates the thickness of the medium crossed by the exciting beam and provides a temporal resolution of 0.07 fs. Delay times t_1 (coherence time between first and second exciting pulse), t_2 (population time between second and third exciting pulse) and t_3 (rephasing time between the third exciting pulse and the emitted signal) are defined.

The outcome of the experiment is a 3D array of data describing the evolution of 2D frequency-frequency correlation maps as a function of t_2. In each map, the excitation and emission frequency axes are obtained Fourier transforming t_1 and t_3, respectively.

Experimental parameters: (i) the pulse duration, optimized through FROG measures (Figure S3), was compressed to about 9 fs. (ii) the exciting energy on the samples was set to about 7 nJ per pulse; (iii) the beam waist was about 100 μm; (iv) the population

time (t_2) was scanned from 0 to 450 fs, in steps of 7.5 fs, while the coherence time (t_1) was scanned from 0 to 125 fs in steps of 3 fs; (v) all the measurements have been performed at ambient temperature; (vi) each experiment was repeated at least five times to ensure reproducibility. Data analysis was performed by exploiting the global fitting methodology described in Ref[4] and time-frequency transform analysis[5].

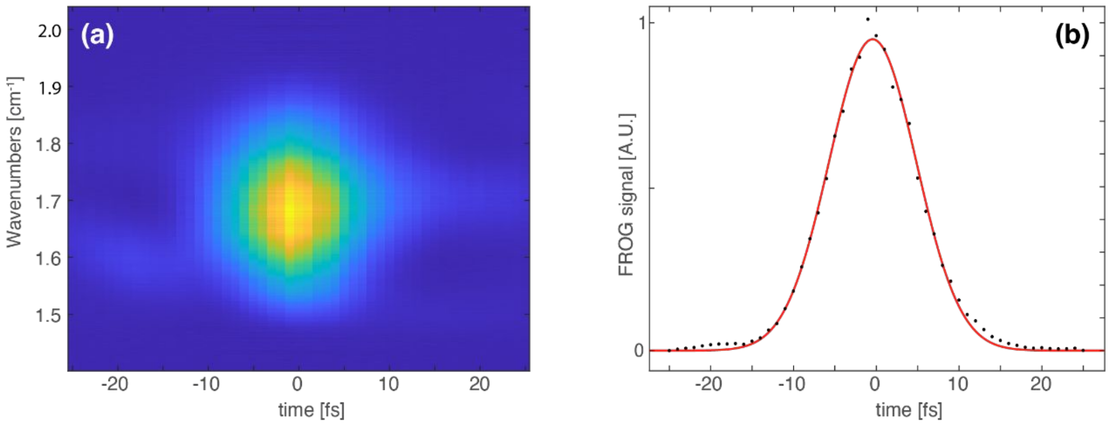

Figure S3. Characterization of the pulse in the 2DES experiments. (a) Frequency Resolved Optical Gating (FROG) measurement and (b) the associated signal integrated along the wavenumber axis. Black dots are experimental points, and the red line is a Gaussian fit. A pulse with FWHM of about 9 fs was obtained.

S2.2 Additional 2DES maps

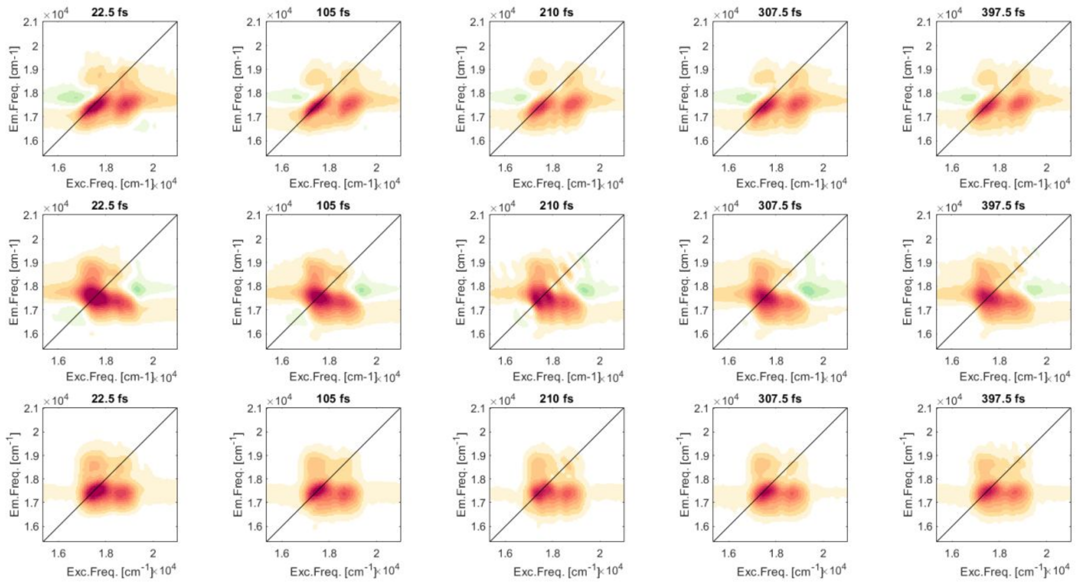

Figure S4: 2DES maps at selected values of population time for the HomoB sample. rephasing (first line); non-rephasing (second line) and purely absorptive (total) signal (third line).

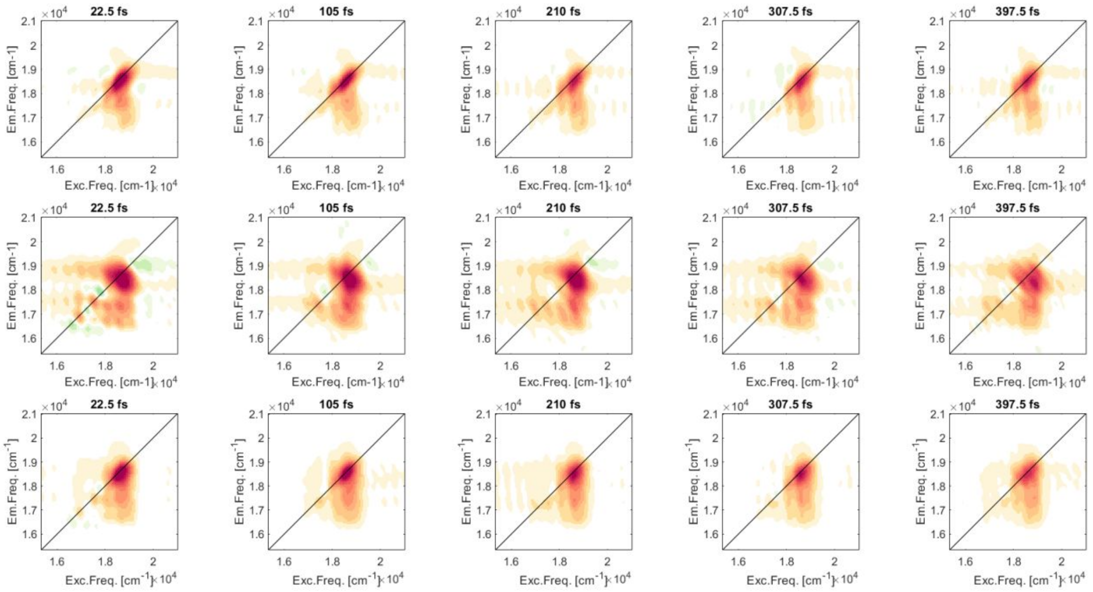

Figure S5: 2DES maps at selected values of population time for the HomoS sample. rephasing (first line); non-rephasing (second line) and purely absorptive (total) signal (third line).

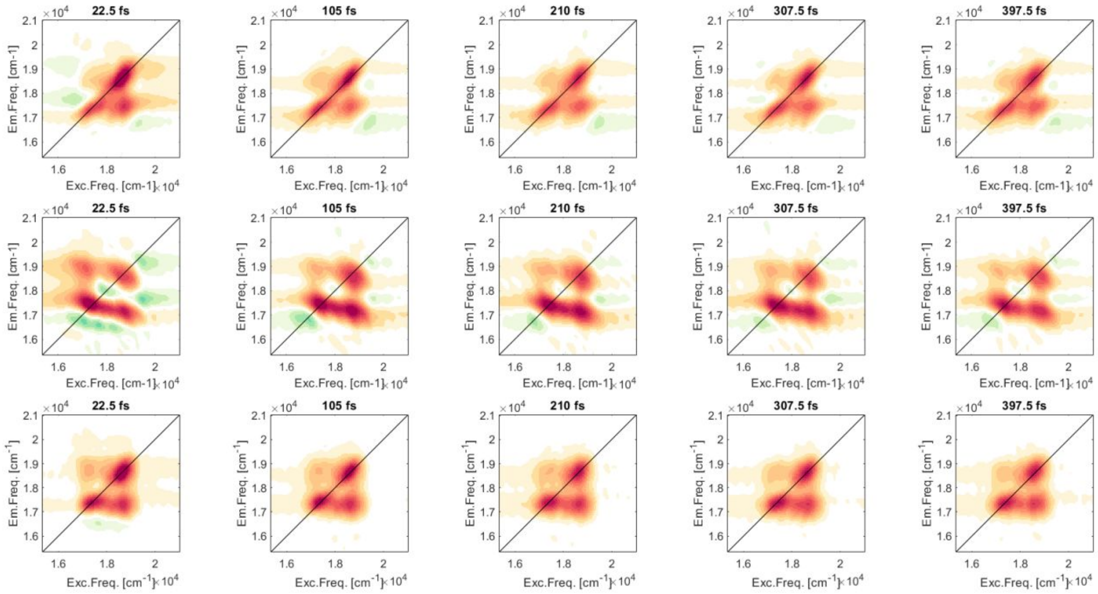

Figure S6: 2DES maps at selected values of population time for the MixC sample. rephasing (first line); non-rephasing (second line) and purely absorptive (total) signal (third line).

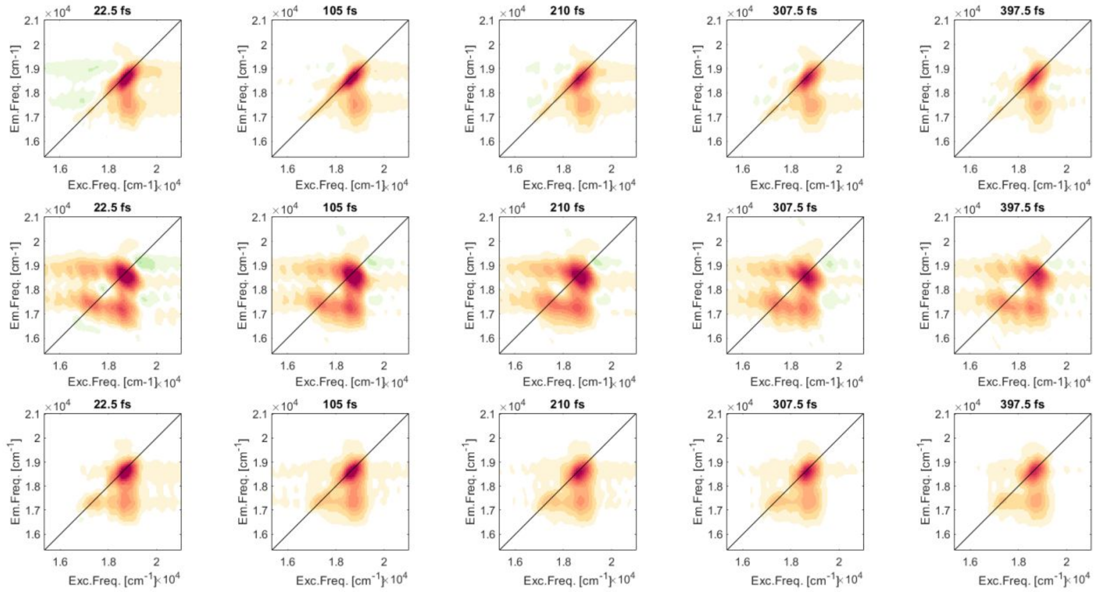

Figure S7: 2DES maps at selected values of population time for the MixO3 sample. rephasing (first line); non-rephasing (second line) and purely absorptive (total) signal (third line).

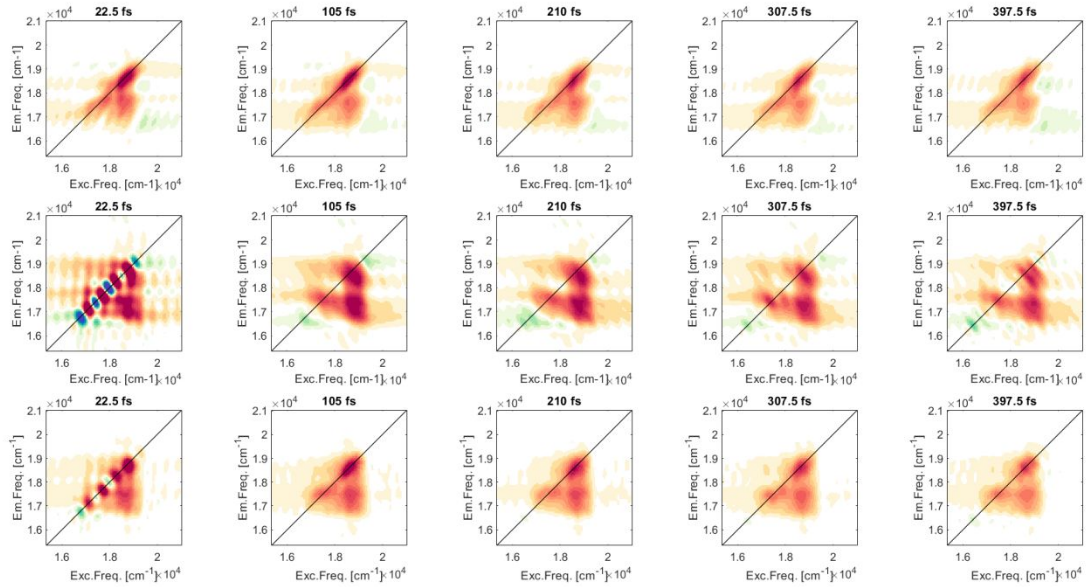

Figure S8: 2DES maps at selected values of population time for the MixO9 sample. rephasing (first line); non-rephasing (second line) and purely absorptive (total) signal (third line).

S2.3. Additional TFT analysis

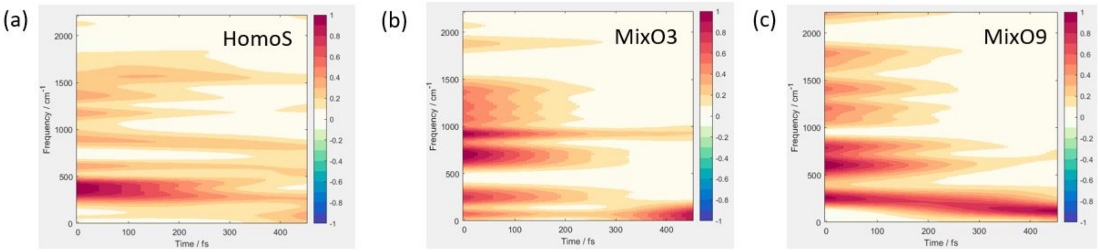

Figure S9: TFT applied for the HomoS (a), MixO3 (b) and MixO9 (c) samples at the signal extracted at off-diagonal coordinates as shown in panel (a) in Figure 5.

S3. Additional details about fluorescence quenching experiments

Emission lifetime decay measurements were performed at Prof. Nir Bar-Gil's lab by time-correlated single photon counting (TCSPC) method. This technique is a statistically significant method, and a high-repetition light source is needed to accumulate enough photonic events for the required statistical data. Excitation achieved by 532 nm pulse laser having pulse duration of 50 ps. The emission was collected by Single Photon Counting Modules (SPCM, single photon detector having instrument response function of 300 ps) having filtering the laser by long pass 625 nm abs filter.

The data was fitted by multiexponential decay model $\sum_{i=1}^{2} a_i \mathrm{e}^{-\frac{t}{\tau_i}}$ -- .

1. Kotov, N. A., Dekany, I. & Fendler, J. H. Layer-by-Layer Self-Assembly of Polyelectrolyte-Semiconductor Nanoparticle Composite Films. *J. Phys. Chem.* **99**, 13065–13069 (1995).
2. Neubauer, A. *et al.* Local Cathode Luminescence Resonant Peak in Hybrid Organic Nanocrystal Systems. *J. Phys. Chem. C* **116**, 15641–15645 (2012).
3. Bolzonello, L., Volpato, A., Meneghin, E. & Collini, E. Versatile setup for high-quality rephasing, non-rephasing, and double quantum 2D electronic spectroscopy. *J. Opt. Soc. Am. B* **34**, 1223–1233 (2017).
4. Volpato, A., Bolzonello, L., Meneghin, E. & Collini, E. Global analysis of coherence and population dynamics in 2D electronic spectroscopy. *Opt. Express* **24**, 24773–24785 (2016).
5. Volpato, A. & Collini, E. Time-frequency methods for coherent spectroscopy. *Opt. Express* **23**, 20040–20050 (2015).
